# Assessment of Toxicity and Biodegradability of Poly(vinyl alcohol)-Based Materials in Marine Water

**DOI:** 10.3390/polym13213742

**Published:** 2021-10-29

**Authors:** Olalla Alonso-López, Sara López-Ibáñez, Ricardo Beiras

**Affiliations:** 1 Grupo ECOTOX (EcoCost) ECIMAT, Centro de Investigación Mariña, Universidade de Vigo, 36331 Galicia, Spain; olallacristina.alonso@uvigo.es (O.A.-L.); salopez@uvigo.es (S.L.-I.); 2 Departamento de Ecoloxía e Bioloxía Animal, Facultade de Bioloxía, Universidade de Vigo, 36310 Galicia, Spain

**Keywords:** poly(vinyl alcohol), glycerol, microplastics, biodegradation, toxicity, marine water

## Abstract

Due to the continuous rise in conventional plastic production and the deficient management of plastic waste, industry is developing alternative plastic products made of biodegradable or biobased polymers. The challenge nowadays is to create a new product that combines the advantages of conventional plastics with environmentally friendly properties. This study focuses on the assessment of the potential impact that polyvinyl alcohol (PVA)-based polymers may have once they are released into the marine environment, in terms of biodegradation in seawater (assessed by the percentage of the Theoretical Oxygen Demand, or % ThOD, of each compound) and aquatic toxicity, according to the standard toxicity test using *Paracentrotus lividus* larvae. We have tested three different materials: two glycerol-containing PVA based ones, and another made from pure PVA. Biodegradation of PVA under marine conditions without an acclimated inoculum seems to be negligible, and it slightly improves when the polymer is combined with glycerol, with a 5.3 and 8.4% ThOD achieved after a period of 28 days. Toxicity of pure PVA was also negligible (<1 toxic units, TU), but slightly increases when the material included glycerol (2.2 and 2.3 TU). These results may contribute to a better assessment of the behavior of PVA-based polymers in marine environments. Given the low biodegradation rates obtained for the tested compounds, PVA polymers still require further study in order to develop materials that are truly degradable in real marine scenarios.

## 1. Introduction

### 1.1. Marine Plastic Pollution

Plastics are one of the most used materials nowadays due to their low cost, light weight, high durability and excellent isolation properties. The annual production in 2019 reached almost 370 million tons [1]. Nevertheless, this high demand and long environmental persistence caused their widespread accumulation into the environment. Plastic is the predominant component of marine litter; it is estimated that around 8 million tons of mismanaged plastic are released into the ocean every year [2]. Environmental weathering of plastics leads to fragmentation into smaller particles, and fractions less than 5 mm are called microplastics [3]. Microplastics pose a potential risk not only for filter feeders, which can end up consuming plastic debris [4] but also for all trophic levels [5]. As particle size decreases, the bioavailability, translocation and toxicity to organisms increases [6].

United Nations Sustainable Development Goals 2015 (SDGs) include, as part of Goal 14, a call to reduce impacts from plastics [7]. Efforts from all around the world are now developing new synthetic materials innocuous for the environment according to the different end-of-life scenarios. This includes regulatory efforts such as the EU initiative to phase out some single-use plastics (Directive 2019/904). Materials based on renewable components and biodegradable options are already being developed as alternatives to conventional polymers. Nowadays, the production of plastics from renewable raw materials is 1% of total plastic production [8]. However, although these new materials are promising, they present new challenges, such as the potential toxicity of the degradation products, or their rapid fragmentation into microplastics [9].

### 1.2. Polyvinyl Alcohol

Poly(vinyl alcohol) (PVA) is a synthetic water-soluble biopolymer that is generally prepared by the saponification of polyvinyl acetate [10]. Remarkable advantages of this potentially biodegradable material are transparency, good mechanical and thermal properties and resistance to oxygen permeation [11].

Products made by PVA constitute the largest volume of water-soluble polymer produced this century [12], with 650,000 tons of PVA per year around the globe [13]. PVA can be found in a wide range of items, including food packaging (31.4% of the demand), construction, electronics, coatings, printing, textile, cosmetics and paper [14]. Likewise, the use of PVA in pharmaceutical and medical applications, including tablets, contact lenses and surgical threads, have been reported [15]. Most of these materials can reach the environment without passing through any integrated system of waste treatment [12]. Another less known use of PVA that implies direct input into the ocean is the equipment for sports fishing, such as bags with dry fishing bait attached to the hook that are eventually released [16].

PVA is perhaps the only fully synthetic C-chain polymer biodegradable in aerobic [17] and anaerobic conditions [18], although in laboratory trials, acclimation in order to allow growth of selected microorganisms is usually essential to achieve rapid degradation [17]. Certain bacteria and fungi contain specific oxidases and dehydrogenases that promote an oxidative reaction of the tertiary carbon atoms of PVA chain forming hydrolyzable b-hydroxylketone and 1,3-diketone groups along the polymer backbone [19,20]. Nevertheless, these microorganisms are not present in most environmental scenarios where degradation rates are very low [21].

### 1.3. Plastic Biodegradation

Biodegradability is a plastic end-of-life option that uses the microorganisms present in a particular environment to completely remove plastic products by mineralization to CO_2_, water and biomass. This process depends on the chemical structure of the polymer, the additives that it contains and the environmental conditions. Usually, the biodegradation of plastics is carried out by scission of the polymer backbone by hydrolysis or enzymatic cleavage initiated by microorganisms that can digest the polymer [22]. Biodegradable plastics can be decomposed on industrial composting facilities [23,24] but many of them are not suitable for biodegradation in natural environments. Previous studies tested the biodegradability of potentially biodegradable plastics in seawater by recording weight loss [25,26,27], oxygen consumption as biological oxygen demand (BOD) or CO_2_ evolution [28,29].

### 1.4. Toxicity of Plastics

Marine pollution analysis was commonly based on the performance of chemical analysis. Nonetheless, due to the high complexity of pollutant mixtures and their interactions with environmental factors, this does not offer sufficient information on their potential effects on organisms in the natural environment. Therefore, the evaluation of toxicity in marine waters must integrate conventional analyses with biological methods such as bioassays [30].

The toxicity of common plastic in the marine environment was previously studied [31,32,33], and frequently associated to chemical additives [34] or sorbed environmental pollutants [33]. Nonetheless, biopolymer toxicity is an emerging issue, since adverse effects of biodegradation products must be taken into account. Some components of water-soluble biopolymers such as ethylene released during degradation can have negative effects on surrounding organisms [35]. On the other hand, some biopolymers, e.g., PHB, seemed to impact aquatic organisms through different mechanisms associated with the higher abundance of plastic particles within the nanometric range found in these resins and absent in other materials [34,36].

The main objective of this research is to provide new insights about the potential risks of microplastics from PVA materials for the marine environment. With that aim, biodegradation studies and ecotoxicological tests were performed in order to have a complete insight into the potential impact of these materials.

## 2. Materials and Methods

### 2.1. Tested Materials

PVA samples were provided by the Plastic Technology Centre AIMPLAS (Spain) from three different stocks (Table 1). Two of them consisted of a mixture of PVA and glycerol, a common plasticizer used in the production of bioplastic [37], and the third one was a plain PVA resin. All the materials were micronized by a ZM200 ultracentrifuge mill (Retsch, Verder Scientific) and sieved through a <250 µm metallic mesh in order to standardize particle size for further testing. A biodegradable material, PHB powder (<250 µm), purchased from Helian Polymers, was used as a reference material.

### 2.2. Biodegradation Study

Biodegradation of the materials was tested following the UNE-EN ISO 14851:2019 [38] for the oxygen demand measurement in a close respirometer, adapted to seawater as follows. NaNO_3_, Na_2_HPO_4_·2H_2_O, and FeCl_3_·6H_2_O purchased from Merck were used as sources of N, P and Fe, and the nutrient formulation was calculated to fit the well-known Redfield ratio 106:16:1:0.1 for C:N:P:Fe [39,40].

The tests were carried out with a set of 500 mL amber glass incubation bottles closed by respirometer units with piezoresistive electronic pressure sensors OxiTop^®^-i IS 6-WTW. The experiments included blanks (with no sample), PHB positive controls and the tested plastic materials, all treatments per duplicate. The medium for the experiment included 0.8 µm filtered seawater sterilized with UV light, enriched with nutrients as explained above, and 1% v. of marine inoculum. The inoculum used was sediment interstitial water collected the same testing day from the beach of Canido (Vigo, NW Iberian Peninsula) in a location under the influence of a freshwater course submitted to frequent discharges of urban wastewater. Then, 250 mL of the medium and 25 mg of microplastic sample were added to each bottle with a magnetic stirrer for continuous mixing. Before closing the bottles, a rubber with KOH pearls was placed in the neck in order to capture the evolved CO_2_. Biological oxygen demand (expressed as mg O_2_/L) was recorded every day for a 28-day period.

Blank-corrected BOD values are expressed as % of the Theoretical Oxygen Demand (ThOD), defined as the theoretical amount of oxygen required to fully degrade the whole organic carbon content of the polymer to CO_2_, as described in UNE-EN ISO 14851:2019 [38], and as percentage of the BOD recorded in the positive control (%C+). The latter is more suited to biopolymers, since the main purpose is to detect relevant biodegradation compared to a well-known biodegradable polymer, and because the actual atomic composition of tested polymers is not always disclosed.

### 2.3. Toxicity Test

Marine aquatic toxicity of PVA leachates was tested using the *Paracentrotus lividus* sea-urchin embryo test (SET) according to [41]. The leachates were obtained in artificial seawater [42] at 10 g/L and tested using geometrical serial dilutions (×1, ×1/3, ×1/10…) until the dilution was found, with no toxic effect (NOEC). Four replicates per treatment and eight controls were added. Sea urchins were provided by the Marine Culture Unit of ECIMAT (CIM-University of Vigo) the same day the tests were performed. Eggs were fertilized with a few µL of sperm in a 50 mL measuring cylinder and transferred before the first division into glass vials with 4 mL of the experimental solution and a density of 40 individuals per mL. Vials were closed with Teflon-lined caps and kept at 20 °C in the dark for 48 h. Afterwards, the incubation time vials were fixed with 40% formalin for ulterior observation under an inverted microscope (Leica DMI 4000B). The length as a maximum linear dimension was measured on 35 larvae per vial using Leica Application Suite LAS image analysis software version 4.12.0 (Leica Microsystems, Germany). The acceptability criteria for this test were the percentage of fertilized eggs (>98%) and size increase in controls >253 µm [43].

Statistical analyses were carried out using IBM SPSS (v. 24). Normal distribution and homoscedasticity were checked using the Shapiro–Wilk and Levene’s tests, respectively. Leachate dilutions that produced larval growths significantly different to the control (*p* < 0.05) were identified using Dunnett’s post hoc test or, when the variances were not homogeneous, Dunnett’s T3 test in order to find the lowest no observed adverse effects concentration (NOEC) and the lowest observed adverse effect concentration (LOEC). The leachate dilutions that produced a 50% decrease in larval growth with respect to the control (EC_50_) was also calculated and their 95% confidence intervals (CIs) were calculated adjusting the data obtained to the Probit dose-response model. Toxic Units (TU) were calculated as TU= 1/EC_50_, and materials were classified following the assessment criteria shown in Table 2.

## 3. Results and Discussion

### 3.1. Biodegradability Test

**Figure 1:**
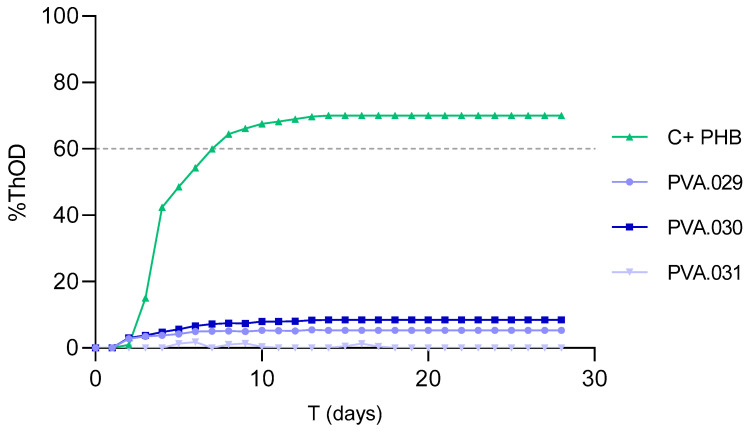
Biodegradation expressed by %ThOD for the different materials and the positive control (C+ PHB). Discontinuous line: 60% biodegradability threshold.

Figure 1 shows the biodegradation results for the 28 days of incubation obtained for each material. According to UNE-EN ISO 14851:2019 guidelines [38], a substance is considered biodegradable if the BOD is higher than 60% ThOD. PHB was used as positive control due to its well-known biodegradability in seawater, as shown by Tachibana et al. who reported 80% biodegradation in 25 days [28]. Similar percentages of biodegradation were found in the present study, where the PHB control achieved 70% degradation after 28 days. Some standards propose an extended incubation period of 60 days, and up to 180 days (ISO 19679, ISO 23977-2, ASTM D7991-15) [44,45]. This extension considerably increases the technical complexity of the experimental setup and increases the chances of failures during the execution of the tests. Moreover, even though we may need to consider a longer period for certain materials, it was observed that the degradation became stable after 28 days for both positive control and tested materials. Materials PVA.029 and PVA.030, including 15% glycerol in their composition, showed a final biodegradation percentage of 5.3 and 8.4% ThOD, respectively, and are thus classified as slightly biodegradable. For the PVA.031 sample, composed by PVA only, we observed that the biodegradation was negligible. However, studies about degradability of PVA on freshwater inoculated with municipal sewage sludge reported percentages of biodegradation of 13% during 21 days [12]. The marine medium used in the present studies (pH 8.3) may lack microbial strains present in wastewater. Additionally, seawater shows a pH > 8, which may have retarded degradation, since biodegradation of PVA was reported to be higher in acidic aqueous solutions than in alkaline ones [46].

Expressed as a percentage of the positive control, the biodegradation rate of the sample PVA.029 was 7.8 and 12.4% for the 030.PVA one, values that still represent a low degree of biodegradation. As expected, the material with the higher degree of hydrolysis (030.PVA) results as the most biodegradable, as reduced molecular weight is a precondition for microbial attack, and the hydrolytic mechanisms enhance the biodegradation processes [47]. Moreover, PVA materials can reach up to 60% degradation in 32 days depending on the degree of solubility of the polymer [48].

The detected increase of 6.85% on average in the biodegradation rate for glycerol-containing PVA may be due to the changes in the hydrophilic characteristics of the glycerol that reduce internal hydrogen bonds in the polymer chain and decrease the residual mass as described by Abdullah and Dong (2019) [49]. They observed an increase of 23.33% in biodegradation rates when adding glycerol. Moreover, raw glycerol has biodegradation on natural water between 68 and 78% [50].

It should be noted that due to the lack of research about biodegradability on microparticles from bioplastics, this study can be only compared to studies using larger fragments (films, pellets, etc.). There is a gap of information in this area that needs to be covered, given that the final faith of microplastics and their degradation products is not well known [51].

Some labels have been developed by industry to distinguish plastics that can biodegrade in the environment. For instance, the label created by Vinçotte OK Biodegradable WATER applies the standard BS EN ISO 14851:2019 [38,52]. Nevertheless, as Harrison et al. pointed out, there is no agreement on which standards to use for plastic biodegradation [53], and a more realistic point of view unifying all the criteria required for these specific materials is needed. It is also important to bear in mind that biodegradability is an intrinsic property of a material, but performed biodegradation will necessarily depend on environmental conditions, and thus no single standard will be able to be representative of the multiple end-of-life scenarios present in the aquatic environments.

### 3.2. Toxicity Test

**Figure 2:**
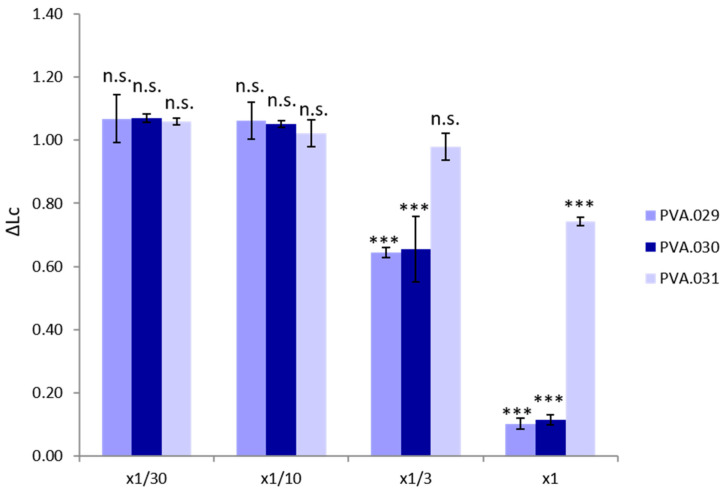
*Paracentrotus lividus* larvae size increase ratio compared to control treatment (ΔLc) for the different sample dilutions. * *p* < 0.05, ** *p* < 0.01, *** *p* < 0.001.

Toxicity of the materials using the standard sea urchin model with a concentration of 10 g/L is reflected in Table 3. According to the current EU classification of chemicals, based on their short-term aquatic toxicity [54], all samples are classified as harmless (EC_50_ > 100 mg/L). Following the TU classification (see Table 2), the polymer composed of plain PVA showed a total absence of toxicity with a TU < 1. This result is supported by toxicity bioassays in seven other aquatic species including marine water individuals *Hyalella azteca* and *Leptocheirus plumulosus* that showed a low toxicity for the PVA material [55]. The materials PVA.029 and PVA.030 presented a certain degree of aquatic toxicity (2.3 and 2.2 TU, respectively) with a larval growth lower than the PVA.031 sample (Figure 2). Consequently, PVA.029 and PVA.030 could be classified as slightly toxic.

ECHA [56] shows a CE_50_ for glycerol on freshwater invertebrate organisms at 48 h of 1995 (1851 to 2068) mg/L, corresponding to a TU < 1. According to studies carried out on three different aquatic bioindicators, glycerol ethers can be classified as harmless for the environment in acute exposure [57]. Glycerol has been presented as a sustainable solvent for its good biodegradability and low toxicity on certain organisms [58], but its effects when blended with polymers and other materials need further research in order to confirm that these compounded materials do not pose a threat for the environment. In fact, we have found a slight but significant increase in toxicity for glycerol-PVA blends, compared to plain PVA.

Similarly, it is necessary to include the assessment of the potential toxicity of degradation products in the scheme of evaluation of biopolymers before they can be presented as more environmentally friendly alternatives compared to conventional plastics.

## 4. Conclusions

This study characterized the biodegradation and ecotoxicity of PVA polymers in marine environments. The results obtained, though limited, support the lack of biodegradability of PVA materials under conditions representative of a natural marine environment. The slight biodegradability of the blended materials was attributable to the glycerol component of the blend. Since plastics of different nature are increasingly found in marine environments, standards with a view to assess biodegradation under realistic marine end-of-life scenarios are urgently needed.

On the other hand, none of the tested polymers pose a relevant risk to the model marine organism used, the sensitive Sea urchin Embryo Test (SET), but slight toxicity arises for glycerol-containing blends. These tests should be conducted with a broader group of aquatic species. These standards should take into account not only biodegradability in terms of mineralization to CO_2_, but also mechanical degradation, potential release of microplastics and lack of toxicity of additives and biodegradation products.

## Figures and Tables

**Table 1:** Characteristics of the materials tested.

Reference	Brand	Hydrolysis, Mole %	Viscosity (cps)	% PVA	% Glycerol
PVA.029	Selvol™ Polyvinyl Alcohol 205	87.00–89.00	5.2–6.2	85	15
PVA.030	Exceval HR 3010	99.0–99.4	12.0–16.0	85	15
PVA.031	PVA KURARAY POVALTM	98.0–99.0	3.2–3.8	100	0

**Table 2:** Toxicity classification based on the sea-urchin embryo test Toxic Units (TU).

EC_50_ (mg/L)	TU	Toxicity
>10,000	<1	None
2000–10,000	1 ≤ TU< 5	Slight
400–2000	5 ≤ TU< 25	Relevant
<400	≥25	High

**Table 3:** Toxicity of different materials expressed as no observed adverse effects concentration (NOEC) and the lowest observed adverse effect concentration (LOEC); toxic units (TU); 50% decrease in larval growth with respect to the control (EC_50_).

Sample	NOEC (g/L)	LOEC (g/L)	TU	CE_50_ (mg/L)
PVA.029	1	3.33	2.3	4285 (1585–12164)
PVA.030	1	3.33	2.2	4382 (3750–5017)
PVA.031	3.33	10	<1	2403.8 (1814.8–3773.5)

## Data Availability

Not applicable.
